# *CLEC4C* p.K210del variant causes impaired cell surface transport in plasmacytoid dendritic cells of amyotrophic lateral sclerosis

**DOI:** 10.18632/oncotarget.7886

**Published:** 2016-03-01

**Authors:** Su Min Lim, Young-Eun Kim, Won Jun Choi, Ki-Wook Oh, Min-Young Noh, Min-Soo Kwon, Minyeop Nahm, Namshin Kim, Chang-Seok Ki, Seung Hyun Kim

**Affiliations:** ^1^ Department of Translational Medicine, Graduate School of Biomedical Science and Engineering, Hanyang University, Seoul, Republic of Korea; ^2^ Green Cross Genome, Yongin, Republic of Korea; ^3^ Department of Neurology, Sheikh Khalifa Specialty Hospital, Ras Al Khaimah, United Arab Emirates; ^4^ Department of Neurology, College of Medicine, Hanyang University, Seoul, Republic of Korea; ^5^ Cell Therapy Center, Hanyang University Hospital, Seoul, Republic of Korea; ^6^ Department of Pharmacology, School of Medicine, CHA University, CHA Bio Complex, Seongnam, Republic of Korea; ^7^ Epigenomics Research Center Genome Institute, Korea Research Institute of Bioscience and Biotechnology, Daejeon, Republic of Korea; ^8^ Department of Laboratory Medicine and Genetics, Samsung Medical Center, Sungkyunkwan University School of Medicine, Seoul, Republic of Korea

**Keywords:** C-type lectin, whole-exome sequencing, dilysine motif, ER retention, amyotrophic lateral sclerosis, Gerotarget

## Abstract

The type II C-type lectin CLEC4C is a transmembrane protein selectively expressed on plasmacytoid dendritic cells (PDCs). Although its mechanism of action remains unclear, triggering of the extracellular C-terminal C-type carbohydrate recognition region of CLEC4C regulates the secretion of proinflammatory cytokines and type I IFNs in PDCs. Applying whole-exome sequencing in a patient with juvenile amyotrophic lateral sclerosis (ALS) and both healthy parents, we identified a *de novo CLEC4C* variant (c.629_631delAGA; p.Lys210del). In this study, we report that the deletion of a lysine residue at the extracellular region of CLEC4C yields a C-terminal dilysine motif that results in endoplasmic reticulum (ER) retention of the protein in transfected HeLa and Jurkat T lymphoma cell models. As a consequence, a decrease in the surface expression of CLEC4C and the ER localization of the mutant construct were observed. Furthermore, depletion of the cell surface CLEC4C level was also observed in the patient PDCs, further suggesting that the variant p.Lys210del CLEC4C may contribute to juvenile ALS susceptibility.

## INTRODUCTION

Amyotrophic lateral sclerosis (ALS) is a lethal neurodegenerative disorder that involves progressive degeneration of both upper and lower motor neurons throughout the brain and spinal cord [1]. The onset of classical ALS presents clinically in late adulthood, whereas the juvenile form of ALS has an onset before 25 years of age, with a milder presentation [2]. A variety of genetic approaches has established the means for rapid discovery of the unsolved genetic and cellular events in ALS. High-throughput DNA sequencing has been used to find the genetic variations underlying sporadic ALS (SALS) (*C9ORF72* repeat expansions, *FUS*, *TARDBP*, *SOD1*, and more) and juvenile SALS (*ALS2*, *SETX*, *SIGMAR1*, and more), as well as additional genes that account for 11% of SALS cases [3–5]. However, the remaining 89% of SALS cases are yet to be identified.

To elucidate previously unknown ALS-causing genetic mutations, we applied whole-exome sequencing in a juvenile SALS patient and healthy parents and identified one *de novo* variant in *CLEC4C* (c.629_631delAGA; p.Lys210del). The *CLEC4C* gene contains 7 exons, located on chromosome 12p13.31, and encodes a member of the C-type lectin (CLEC) domain family with 213 residues [6]. CLEC4C is the specific marker confined to human plasmacytoid dendritic cells (PDCs). PDCs, a subset of DC, are derived from bone marrow progenitor cells that traffic from peripheral blood to lymphoid organs and the Central Nervous System (CNS) [7–9]. PDCs produce cytokines to bridge the innate and adaptive immune responses, present antigens to activate T cells, and induce the cytotoxicity and tolerance involved in immune responses [10, 11]. In response to viral infections, PDC endocytoses and delivers viral particles to endosomes containing Toll-like receptors 7 (TLR7) and TLR9 [10]. After the engagement of TLRs, the cell produces large amounts of type I interferons (IFNα and IFNβ) and other proinflammatory cytokines. However, ongoing activation of PDCs and IFNα overproduction has been reported to cause inflammatory diseases such as systemic lupus erythematosus (SLE) and psoriasis; therefore, regulatory systems are needed to counteract the sustained secretion of cytokines [12, 13]. Although its mechanism of action is yet to be established, triggering the extracellular C-terminal C-type carbohydrate recognition domain of CLEC4C interferes with the sustained secretion of TLR9-mediated cytokines and is responsible for regulating the production of TLR-induced cytokines in PDCs [6, 14].

Plasma membrane proteins such as CLEC4C are processed from the endoplasmic reticulum (ER) and are delivered downstream to the plasma membrane. However, the membrane proteins with a C-terminal dilysine motif KKXX or KXKXX employ an ER retention mechanism that targets and traps them in the ER [15–17]. The patient in this study possess a deletion variant (p.Lys210del) in CLEC4C, resulting in a loss of a highly conserved lysine and a gain of a C-terminal dilysine motif potentially involved in ER retention. Herein, we investigated the cellular properties of the deletion CLEC4C mutant yielding the dilysine motif in the transfected HeLa and Jurkat cells, and the patient's PDCs. Extracellular dilysine motif at the C-terminal region of CLEC4C disturbs the cell surface expression of the protein and results in ER retention. These findings suggest that lack of surface expression of CLEC4C may be one of the genetic pathophysiological features in ALS.

## RESULTS

### Clinical findings and genetic study in a juvenile sporadic ALS patient

**Figure 1:**
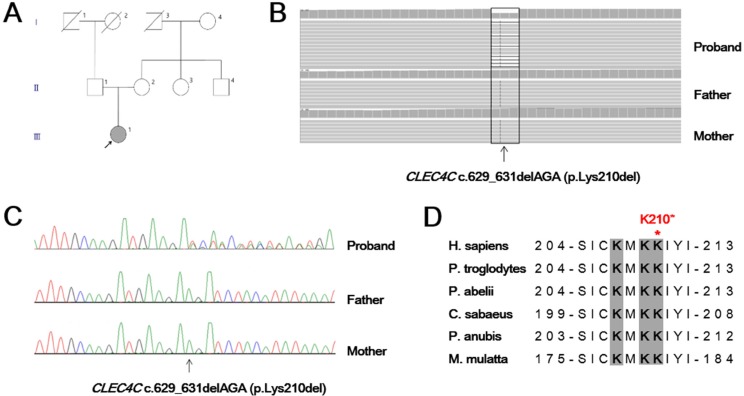
Genetic analysis of a juvenile ALS patient (**A**) Family pedigree of the juvenile amyotrophic lateral sclerosis patient family shows the *de novo* c. 629_631delAGA mutation in the participating individual (arrow). (**B**) Exome sequencing of the proband with the c.629_631delAGA (p.Lys210del) and parents. Variant reads of *CLEC4C* c.629_631delAGA were 53% (64/122) of total reads in the proband, suggesting heterozygosity of the allele. (**C**) Sanger sequencing of the *CLEC4C* gene in the proband confirmed a *de novo* occurrence of heterozygous AGA deletion at nucleotide position from 629 to 631. (**D**) Amino acid sequence alignment of the CLEC4C protein from different species. The regions in grey, shown in bold, are predicted to have a polybasic motif and are highly conserved. The location of the p.Lys210del (p.K210*) variant near the C-terminal end is demonstrated with a red asterisk. H. sapiens, human; P. troglodytes, chimpanzee; P. abelii, orangutan; C. sabaeus, green monkey; P. anubis, olive baboon; M. mulatta, rhesus monkey.

We performed whole-exome sequencing using Illumina HiSeq 2000 in a juvenile SALS patient and both healthy parents (Figure 1A). From this analysis, we identified a novel in-frame deletion variant (c.629_631delAGA; p.Lys210del) in the *CLEC4C* gene, which was confirmed as *de novo* occurrence by Sanger sequencing (Figure 1B, 1C). This variant was not present in dbSNP141, 1000 Genome Project and Exome Aggregation Consortium. The patient was 20 years old and presented with a twenty-month history of left hand weakness, which progressed slowly to the proximal muscles and spread to the left lower limb for two years. She had no other neurological diseases or family history of neuromuscular disorders. On examination, a weakness with atrophy and fasciculation of the upper and lower limbs was observed. Her deep tendon reflexes were very brisk in the upper and lower limbs, and jaw jerk was increased. Ankle clonus and Hoffmann signs were present bilaterally. The revised ALS functional rating scale (ALSFRS-R) was 46 at the first visit. After 21 months, the ALSFRS-R declined to 26.

### The dilysine motif in 210delK CLEC4C mediates ER retention in HeLa cells

CLEC4C is located on the cell surface and consists of an extracellular C-terminal C-type carbohydrate recognition domain, a transmembrane region, and a short N-terminal cytoplasmic tail without an obvious signaling motif [6]. The C-terminal lysine cluster with the sequence 207-KMKKIYI-213 are functionally important residues that are evolutionarily highly conserved in CLEC4C across species including *Homo sapiens* and other mammals (Figure 1D). The patient in this study has the p.Lys210del variant, resulting in a transition from the 207-KXKKXXX-213 polybasic motif, playing a key role in plasma membrane localization [18], to 207-KXKXXX-212 dilysine motif, potentially involved in ER retention. To address whether the loss of a highly conserved lysine in the patient CLEC4C retains the protein in the ER, wildtype and mutant CLEC4C constructs were expressed in human cell lines.

**Figure 2:**
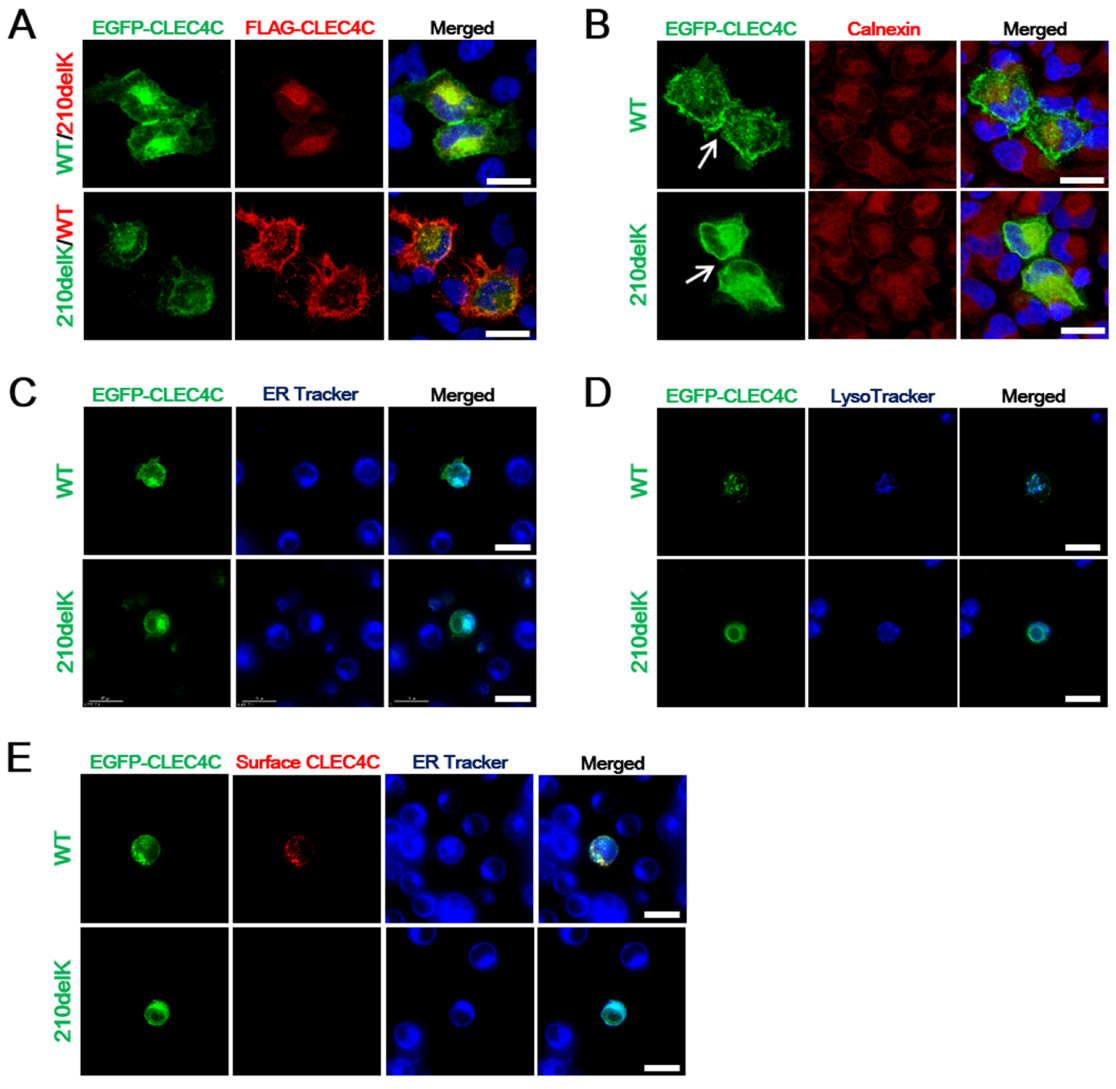
The ALS-associated CLEC4C mutant is deficient in surface expression (**A**) Confocal fluorescent microscopy showing the cellular localization of EGFP-CLEC4C or FLAG-CLEC4C mutants in HeLa cells. Compared to wildtype CLEC4C constructs, mutant CLEC4C demonstrated reduced cell surface transport in HeLa cells. DNA is identified by DAPI. Scale bars: 25 μm. (**B**) Anti-calnexin antibodies were used to label the ER (red) localization of mutant EGFP-CLEC4C in HeLa cells. The two adjacent transfected cells formed extracellular interactions in wildtype CLEC4C, but not in the mutant construct (arrows). DNA is identified by DAPI. Scale bars: 25 μm. (**C**) Live imaging of Jurkat cells transfected with EGFP-CLEC4C wildtype or mutant. Co-localization of ER Tracker Blue-White DPX (ER-specific probe) and EGFP-CLEC4C mutant demonstrate deficient surface expression and localization of the mutant in the ER. Scale bars: 15 μm. (**D**) Mutant CLEC4C in Jurkat cells does not localize with lysosomes. The subcellular localization of CLEC4C was analyzed by LysoTracker for lysosomes (blue). Scale bars: 15 μm. (**E**) Surface CLEC4C staining of wildtype and mutant CLEC4C-expressing Jurkat cells were color coded (green = EGFP-CLEC4C wildtype or mutant. Red = cell surface CLEC4C). ER Tracker Blue-White DPX is used as an index of ER retention in the mutant CLEC4C. Scale bars: 15 μm. All data are presented from representative experiments and each image is representative of a field (*n* = 5) of one of three or four independent experiments. Each experiment was independently repeated three times for A and B or four times for C, D, and E.

Since HeLa cells do not express CLEC4C, we transfected HeLa cells with wildtype or mutant CLEC4C constructs. The constructs encoding either EGFP- or FLAG-tagged wildtype or mutant CLEC4C were co-transfected to eliminate the possible effects of size or type of expression pattern of each tag. In cells expressing either EGFP- or FLAG- tagged wildtype CLEC4C construct, the protein was expressed both on the cell surface and in the cytoplasm (Figure 2A). On the other hand, HeLa cells expressing p.Lys210del CLEC4C with either protein tag had less surface expression of CLEC4C. To determine if the p.Lys210del CLEC4C variant with the polybasic motif changed to a dilysine motif is potentially involved in ER retention, the transfected cells were stained with an anti-calnexin (ER) antibody. As expected, the ring-like pattern of EGFP-CLEC4C (p.Lys210del) around the nuclei was co-localized with ER staining, indicating that p.Lys210del CLEC4C is trapped in the ER and that p.Lys210del of CLEC4C yields a C-terminal dilysine motif (KXKXXX) leading to ER retention (Figure 2B). Interestingly, the transfected cell adjacent to another transfected cell formed a seal between the cell surfaces in wildtype CLEC4C only, suggesting that extracellular domains of these proteins form interactions with each other.

### The dilysine motif in p.Lys210del CLEC4C mediates ER retention in live jurkat cells

CLEC4C is a marker uniquely expressed in human plasmacytoid dendritic cells (PDCs). PDCs are present in peripheral blood and migrate to lymphoid organs and the CNS [7–9]. To elucidate whether the dilysine motif of CLEC4C can mediate ER retention in a more disease-relevant target cell type, we transfected Jurkat T cells. Similar to PDCs, Jurkat is a suspension cell line established from the peripheral blood. We examined the localization of EGFP-tagged CLEC4C wildtype or mutant in Jurkat cells. As shown by HeLa cell transfection, wildtype CLEC4C was expressed in both the cell membrane and the cytosol, while the C-terminal dilysine motif in the mutant prevented the surface expression of CLEC4C in the Jurkat cells. The perinuclear staining of p.Lys210del CLEC4C co-localized with the ER (ER Tracker Blue-White DPX), but not with lysosomes (LysoTracker Blue) (Figure 2C, 2D, respectively). To confirm the surface expression of CLEC4C and surface depletion of p.Lys210del CLEC4C, live cell imaging of the transfected Jurkat cells was performed without fixation or permeabilization process, allowing us to compare surface CLEC4C expression level within live cells. The transfected Jurkat cells were imaged alive after the addition of the primary and the fluorescently labeled secondary antibodies. As expected, the surface expression of wildtype CLEC4C transfected cells, but not the mutant, were detected and labeled (Figure 2E). ER retention of the p.Lys210del CLEC4C construct was confirmed by co-localization of the intracellular EGFP signal with the ER Tracker.

### Impaired cell surface transport of endogenous CLEC4C in the patient PDCs

**Figure 3:**
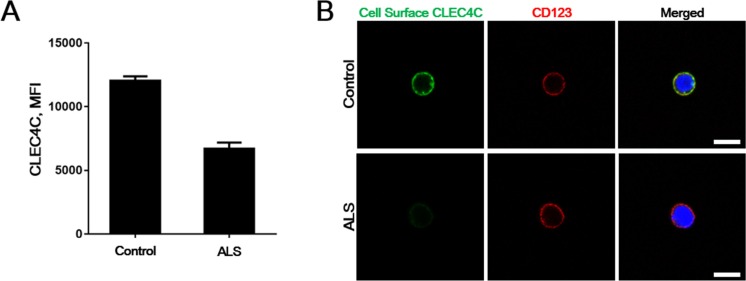
Reduced surface CLEC4C in the PDC of the ALS patient (**A**) A representative control and the ALS patient PDCs were purified and stained for CLEC4C. Mean fluorescent intensity (MFI) of the CLEC4C-positive population are shown from duplicate assays. (**B**) Surface expression of both healthy control and the patient endogenous CLEC4C were detected by anti-CLEC4C antibodies (green). Anti-CD123 antibodies were used to prove PDC-selective cell surface labeling. Reduced surface CLEC4C was observed in the ALS patient. Nuclei are identified by Hoechst dye staining. Scale bars: 7.5 μm. The data are from one experiment using representative PDCs from four healthy controls and one patient.

To test whether the PDC from the juvenile SALS patient with endogenous p.Lys210del CLEC4C demonstrates similar defects in cell surface CLEC4C expression as shown in other cell models, we cultured primary PDC isolated from healthy controls and the patient blood. We sort-purified PDC from PBMCs under sterile conditions by using a PDC isolation kit and FACS (fluorescent-activated cell sorting). Consistent with less surface expression of the p.Lys210del mutation on transfected HeLa and Jurkat cell model, the patient endogenous CLEC4C of PDCs showed less cell surface fluorescent intensity compared to healthy control, displayed by decreased mean fluorescent intensity (MFI) (Figure 3A). This data was confirmed with fluorescent CLEC4C antibodies for cell surface CLEC4C labeling, along with anti-CD123 antibodies for PDC-specific labeling. As expected, the cell surface CLEC4C expression was reduced in the SALS patient PDCs carrying the CLEC4C (p.Lys210del) variant compared to control PDCs (Figure 3B). Thus, the C-terminal dilysine motif on CLEC4C proteins resulting in reduced cell surface transport was confirmed in the patient PDCs.

## DISCUSSION

This is the first report on the reduced surface expression of CLEC4C (p.Lys210del) on PDC in ALS. Our cell modeling results with HeLa, Jurkat, and human PDCs suggest that a novel *de novo* variant p.Lys210del CLEC4C found in a juvenile SALS patient by whole-exome sequencing shows different cellular properties from wildtype CLEC4C constructs or CLEC4C proteins from healthy control cells. The variant p.Lys210del CLEC4C yields a transformation of the polybasic motif of the protein to a dilysine motif potentially involved in ER retention, which results in impaired cell surface transport of the CLEC4C protein.

A link between genetic variation and neuroinflammation has recently been discussed in neurodegenerative diseases, as has disease causing genetic mutations been revealed using whole-exome sequencing approaches. Loss of function mutations in the triggering receptor expressed on myeloid cells 2 (*TREM2*) gene or DNAX-activating protein of 12 kDa (*DAP12*) gene deletions cause polycystic lipomembranous osteodysplasia with sclerosing leukoencephalopathy [also known as Nasu-Hakola disease (NHD)], a rare disease characterized by ankle pain and swellings [19, 20]. Recent studies, however, have implicated that *TREM2* variant is also a risk factor of Alzheimer disease, frontotemporal dementia, Parkinson disease, and more recently, ALS, due to impaired cell surface transport of the protein [21–24]. The study demonstrating a *TREM2* variant as a potent risk factor for ALS is the first genetic influence on identifying a link between neuroinflammation and ALS progression. Likewise, the presented data of a *CLEC4C* variant in our study may also support that the genetic etiology have implications for the neuroinflammation in ALS.

Inflammatory processes in ALS are characterized by microglial, astroglial, macrophage, and dendritic cell (DC) activation in the spinal cord. The ALS patients with more rapid disease progression have increased DC activation in post-mortem spinal cord tissue [25]. DC facilitate antigen presentation to induce effective immune responses upon activation. Their antigen capture and presentation abilities are largely mediated by the cell surface expression of CLEC receptors [26]. Triggering extracellular regions of CLEC4C, which is confined to PDCs, is required to inhibit the overproduction of TLR-induced cytokines in PDCs. Hence, the expression of the cell surface CLEC4C protein is crucial. However, the p.Lys210del CLEC4C variant results in reduced expression of cell surface CLEC4C protein, which results in a consequent loss in the crosslinking of CLEC4C that might alter the levels of cytokines in the patient PDCs, such as the levels of IFNα, IL-6 and TNFα. Contributing to a relatively small number of PDCs purified from PBMC and to prevent burdening in the patient with large amount of blood sampling, a transgenic animal model over-expressing the p.Lys210del CLEC4C variant will be needed for further validation. Nevertheless, this is an initial step towards identifying ALS-associated immune abnormalities leading to SALS.

ALS-associated inflammation and immune abnormalities involving CLEC family have been found in both post-mortem spinal cord tissue from human ALS patients and in animal models of ALS. The overexpression of CLEC4A, within the same CLEC family as CLEC4C, has been identified in SALS postmortem spinal cord, which suggests that CLEC may be associated with ALS pathology [27]. In an animal model, the rat antigen presenting lectin-like receptor gene complex (Aplec) contains seven different *CLEC* gene clusters that regulate motor neuron survival after damage to the spinal nerve [28, 29]. Considering that CLEC4A is overexpressed in SALS patients and that the motor neuron survival is regulated by the Aplec *CLEC* gene cluster in rats after spinal cord injury, CLEC4C may also have a potential role in motor neuronal vulnerability. Our data shown in a juvenile onset ALS patient with a *de novo* variant of the *CLEC4C* gene could be an important initial clue toward understanding the innate immunity defect in pathogenic mechanism of ALS. Thus, further extensive investigation will be needed to determine the mechanism of CLEC4C in PDC and its potential role in ALS susceptibility.

## MATERIALS AND METHODS

### Patient and control sample collection

Among affected individuals diagnosed with ALS at Hanyang University Hospital, Seoul, South Korea, one affected juvenile onset ALS patient participated in this study. The study protocol was approved by the Institutional Review Board of Hanyang University Hospital. Informed written consent was obtained from the patient and her parents and blood samples were collected from the family members.

### Exome sequencing and genetic variation analysis

After obtaining informed consent, genomic DNA from the patient and her parents were prepared from peripheral blood leukocytes using a Wizard Genomic DNA Purification kit (Promega). Whole exome capture and sequencing were performed using an Agilent SureSelect Human All Exon 50 Mb kit (Agilent Technologies) and the HiSeq2000 (Illumina) sequencing platform, respectively. The raw sequence reads were processed and aligned to the GRCh37/hg19 human reference sequence with the Burrows-Wheeler Aligner version 0.7.10 program (http://bio-bwa.sourceforge.net). Duplicate reads were removed with Picard version 1.114, and local alignment optimization was performed with the Genome Analysis Toolkit. After finding a *de novo CLEC4C* variant, Sanger sequencing validation of the *CLEC4C* variant was performed using the BigDye Terminator Cycle Sequencing Ready Reaction kit (Applied Biosystems) on an ABI3100 Genetic Analyzer (Applied Biosystems).

### Cell proliferation

HeLa cells were cultured in Dulbecco's modified Eagle's medium containing 10% fetal bovine serum (Gibco), sodium bicarbonate, sodium pyruvate (Sigma), and antibiotics. Jurkat (T cell leukemia) cells were cultured in advanced RPMI 1640 (Invitrogen) with 10% fetal bovine serum and antibiotics. For PDC culture, human PBMC were isolated from whole blood by density gradient centrifugation using Ficoll-Paque PLUS (GE Healthcare). PDCs were magnetically sorted and isolated using the Plasmacytoid Dendritic Cell Isolation Kit II and LD columns as instructed (Miltenyi Biotec). The sorted cells were > 98% PDCs based on CLEC4C-APC and CD123-PE (both Miltenyi Biotec) staining.

### Plasmids and transfection

N-terminally FLAG- or EGFP-tagged wildtype human *CLEC4C* cDNA was cloned into the pReceiver vector (Genecopoeia). To make the *CLEC4C* (c.629_631delAGA) mutant DNA, *in vitro* mutagenesis was carried out using the EZchange^™^ site-directed mutagenesis kit (Enzynomics). HeLa cells were transiently transfected with FLAG- and/or EGFP-tagged wildtype or mutant human *CLEC4C* cDNA constructs using Lipofectamine 2000 (Invitrogen). For live cell images, Jurkat cells were transfected with EGFP-tagged CLEC4C wildtype or mutant using Lipofectamine 2000.

### Immunofluorescence

Immunofluorescence staining of HeLa cells and PDC from the ALS patient with the CLEC4C (p.Lys210del) mutation and neurologically normal controls was performed as follows. HeLa cells were planted on glass coverslips. After 48 hrs of transfection, the cells were fixed in 4% paraformaldehyde (PFA) for 15 min and permeabilized in 0.3% Triton X-100 for 10 min. The blocking and antibody incubations were performed using a buffer containing 1× PBS and 5% and 2% normal goat serum (Vector Labs), respectively. For immunostaining, antibodies reactive to FLAG (clone M2, mouse IgG1, Sigma-Aldrich) and calnexin (mouse IgG1, BD Transduction Laboratories) were applied. The anti-mouse secondary antibodies were conjugated with Alexa Fluor-488 or tetramethylrhodamine B isothiocyanate (TRITC) (Invitrogen). Surface expression of CLEC4C in PDC was labeled with anti-CD303 (CLEC4C) (clone AC144, mouse IgG1) and anti-CD123 (clone AC145, mouse IgG2a) (both Miltenyi Biotec). The cells were centrifuged and fixed in 4% PFA. During the fixation, the cell nuclei were stained live with 1 μg/ml Hoechst 33342 dye (Invitrogen/Molecular Probes).

### Live cell analysis

For live cell imaging, Jurkat cells were grown in imaging dishes (Chamber slide Lab-Tek II 4; Fisher). The cells were transfected 18 hrs before the experiment. The cells were loaded with 1 μM ER Tracker Blue-White DPX or LysoTracker Blue-DND-22 (Invitrogen/Molecular Probes) to label the ER or lysosomes, respectively. Surface staining of wildtype or mutant EGFP-tagged CLEC4C-expressing cells was performed 15 min prior to imaging. Pure anti-CD303 (clone AC144, mouse IgG1, Miltenyi Biotec) was applied to transfected live Jurkat cells to detect the surface expression of CLEC4C in both wildtype and mutant constructs. Ten minutes after the addition of the primary antibody, anti-mouse secondary TRITC was added for 5 min and the cells were monitored. The cells were imaged live in growing medium using a DeltaVision fluorescent microscopy system (Applied Precision). Images were taken for 15 min, 1 frame every 1 min.
